# Is the Immunization of Pregnant Women against COVID-19 Justified?

**DOI:** 10.3390/vaccines9090970

**Published:** 2021-08-30

**Authors:** Nicola Principi, Susanna Esposito

**Affiliations:** 1 Università degli Studi di Milano, Via Festa del Perdono 7, 20122 Milan, Italy; nicola.principi@unimi.it; 2 Pediatric Clinic, Pietro Barilla Children’s Hospital, Department of Medicine and Surgery, University of Parma, Via Gramsci 14, 43126 Parma, Italy

**Keywords:** COVID-19, COVID-19 vaccine, maternal immunization, mother-to-child transmission, pregnancy

## Abstract

Maternal immunization against some infectious diseases can offer significant advantages for women, preventing maternal morbidity and mortality, or for offspring, preventing fetal disease and conferring passive immunity to neonates. Recently, clinical trials specifically to evaluate the immunogenicity, safety, and tolerability of some of the available coronavirus disease 2019 (COVID-19) vaccines in pregnant and lactating women have been planned, initiated and, in some cases, completed. This paper discusses whether the immunization of pregnant women against COVID-19 is justified and presents knowledge about the immunogenicity and safety of mRNA COVID-19 vaccines for these subjects. The results of recent studies indicate that pregnant women are at increased risk of developing severe disease compared with nonpregnant women of the same age. Studies carried out with mRNA vaccines indicate that the immunogenicity, safety and tolerability of these preventive measures in pregnant women are not different from those in nonpregnant women of the same age. Moreover, antibodies are efficiently transferred through the placenta and can be detected in breastmilk, suggesting a potential prevention of infection in the child. All these findings authorize the use of mRNA vaccines in pregnant women to protect both the mother and the child. However, further studies with larger sample size and with follow-up of the pregnant women vaccinated during different periods of pregnancy and their children are needed to better characterize the immune response of pregnant women, to define when these vaccines should be administered to obtain the best protection, and to measure vaccine efficacy against virus variants in both mothers and infants. COVID-19 vaccines based on different technological platforms cannot presently be used, and their role in pregnant women should be clarified.

## 1. Background

Maternal immunization against some infectious diseases can offer significant advantages for women, preventing maternal morbidity and mortality, or for offspring, preventing fetal disease and conferring passive immunity to neonates. In some cases, both of these goals are achieved, as in the case of influenza [1,2]. The potential of maternal immunization in protecting young infants was made evident by tetanus vaccination during pregnancy contributing to the reduction in incidence of neonatal tetanus [1,3]. This has also become evident by the decrease in the incidence of severe pertussis disease in young infants in countries that have implemented pertussis immunization programs in pregnancy [1,3]. Other vaccines are recommended to pregnant women in outbreak contexts (i.e., yellow fever vaccine), when there is a threat of exposure (i.e., hepatitis A and B, meningococcus, Japanese encephalitis), or when post-exposure prophylaxis is needed (i.e., anthrax, rabies, smallpox) [4,5]. Finally, vaccines that are recognized as potentially effective in reducing common and sometimes severe infectious diseases in pregnant women and/or neonates as well as infants, such as a respiratory syncytial virus (RSV) vaccine and a group B *Streptococcus* (GBS) vaccine, are in an advanced stage of development [6].

However, despite the evidence that vaccines can play a relevant role in pregnant women and their offspring, historical resistance to the development and use of vaccines in these subjects still exists [7]. Until recently, the prevailing ethical approach for immunization during pregnancy was based on the precautionary principle, which limits introduction of a new intervention whose ultimate effects are uncertain. Safety of vaccines administered during pregnancy needs to be evaluated for both the mother and her newborn, and is an important consideration for the mothers’ willingness to receive a vaccine during pregnancy [1]. There is a significant bulk of evidence to support the safety of immunization with tetanus toxoids, the longest standing vaccine recommended during pregnancy [1]. There is also an increasing body of evidence to support the safety of pertussis and influenza immunization during pregnancy [1]. Generally, new vaccines are not designed for use during pregnancy; pregnant women are not included in the initial vaccine research; and studies about the efficacy, safety, and tolerability of vaccines in pregnant women are carried out only when there is already substantial evidence that the vaccines could be potentially useful for the mother–child dyad or at least for one of them [8]. What has been documented during the current coronavirus disease 2019 (COVID-19) pandemic is representative in this regard. Within the first months after the declaration of the pandemic, several reports had already suggested that COVID-19 could have a more serious course in pregnant women, that the pregnancy of severe acute respiratory syndrome coronavirus 2 (SARS-CoV-2) positive women could be burdened by a higher number of complications, and that even the offspring could have a greater risk of clinical problems [9,10,11]. Unfortunately, no importance has been attributed to these findings, and pregnant women are still systematically excluded from all the trials specifically devoted to the evaluation of the efficacy, safety, and tolerability of the COVID-19 vaccine preparations in development. Only recently, after several experts [12,13] and some national institutions [14,15] recommended the use of vaccines in pregnant and lactating women, were clinical trials specifically to evaluate the immunogenicity, safety, and tolerability of some of the available COVID-19 vaccines in pregnant and lactating women planned, initiated and, in some cases, completed. Moreover, more substantial data on the impact of COVID-19 on pregnant women and their offspring have been collected [16,17,18].

Presently, a definitive answer can be given to the question of whether pregnant women should receive the COVID-19 vaccine. Moreover, although preliminary, there are data that seem to indicate that the immunogenicity and safety of at least mRNA COVID-19 vaccines are quite similar to those previously demonstrated in nonpregnant women of childbearing age [19,20]. This paper discusses whether the immunization of pregnant women against COVID-19 is justified and presents knowledge about the immunogenicity and safety of mRNA COVID-19 vaccines in these subjects.

## 2. Factors Supporting the Immunization of Pregnant Women against COVID-19

Pregnancy occurs in a period of life during which SARS-CoV-2 infection is generally asymptomatic or associated with mild disease. Because of this, it is suggested that these vaccines are given to young, healthy adults only after older people and the population of any age at risk, because of more frequent exposure to the virus or suffering from a chronic severe disease, have already been immunized [21,22,23]. To strongly recommend early vaccination of pregnant women regardless of whether or not they have an underlying diseases, evidence should be available that pregnant women infected by SARS-CoV-2 are at increased risk of developing a more severe disease and/or that pregnancy can be characterized by a higher incidence of complications and/or that the fetus and/or the neonate can have a number of relevant clinical problems.

### 2.1. COVID-19 in Pregnant Woman

#### 2.1.1. Disease Severity

During the pregnancy-puerperal period, the structure of the respiratory tract and immune system functions physiologically change, making pregnant women generally more susceptible to respiratory infections and more prone to develop severe disease [24,25]. However, regarding SARS-CoV-2 infection, available data do not support the hypothesis that this infection is more common among pregnant women than in the healthy general population of the same age [26,27]. In contrast, the increased risk of more severe COVID-19 in pregnant women is well demonstrated. Although some of the retrospective studies carried out at the beginning of the pandemic have suggested that COVID-19 in pregnancy could be analogous to the disease diagnosed in the healthy general population [28,29], most of the evaluations later performed comparing carefully selected infected pregnant women with adequately matched noninfected controls have clearly evidenced that pregnancy is a risk factor for the development of severe COVID-19 [30,31,32,33,34].

The increase in the risk of severe disease varied significantly among studies in relation to the baseline characteristics of enrolled individuals and the criteria used to hospitalize pregnant women and to classify disease severity at presentation or later. However, the general conclusions were that, when infected by SARS-CoV-2, pregnant women had an increased risk of being hospitalized, admitted to the intensive care unit (ICU), and treated with ventilatory support, and of dying. A study performed in the USA from 22 January to 3 October 2020, on 409,462 women of reproductive age with symptomatic COVID-19, among whom 23,434 were pregnant and 386,028 were not pregnant [35], showed that although very severe disease was relatively uncommon in both groups, the incidence rates of ICU admission, invasive ventilation and death were significantly higher in pregnant women than in nonpregnant women. ICU admission was needed in 10.5 vs. 3.9 per 1000 cases (adjusted risk ratio (aRR) 3.0; 95% confidence interval (CI) 2.6–3.4), invasive ventilation was needed in 2.9 vs. 1.1 per 1000 cases (aRR, 2.9; 95% CI, 2.2–3.8), and death occurred in 1.5 vs. 1.2 per 1000 cases (aRR, 1.7; 95% CI, 1.2–2.4). These and other similar findings have been recently confirmed by a prospective, well-conducted analysis, the Intercovid Multinational Cohort Study [16]. This study was carried out from March to October 2020 in 43 institutions sited in 18 countries and enrolled a total of 2130 pregnant women, 746 with COVID-19 and 1424 without COVID-19, who were enrolled and followed until hospital discharge. To minimize the risk of bias, all the women had similar demographic characteristics. Moreover, two controls of similar gestational age (±2 weeks) receiving standard antenatal care were concomitantly enrolled immediately after the identification of each infected woman. Among these women, most (85.8%) had a laboratory-confirmed diagnosis, and 38.5% were asymptomatic. Compared to subjects without SARS-CoV-2 infection, pregnant women with a COVID-19 diagnosis had an approximately 22 times increased risk of death. Eleven (1.6%) of them died, compared to only one in the group of women without infection (RR, 22.3; 95% CI, 2.88–172). The need for admission to the ICU/high-dependency unit and referral to a higher level of care were also significantly greater, with RRs of 5.04 (95% CI, 3.13–8.10) and 6.07 (95% CI, 1.23–30.01), respectively. Once admitted to the ICU, women with a COVID-19 diagnosis stayed 3.73 (95% CI 2.37–5.86) days longer than women without SARS-CoV-2 infection.

#### 2.1.2. Pregnancy Complications

Mothers with SARS-CoV-2 can have not only more severe COVID-19 but also a more complicated pregnancy, leading to severe clinical problems for the mother and the conceptus. Most of the data from studies that have evaluated the pregnancy course in SARS-CoV-2-infected women have been collected during the late second or third trimester of pregnancy, and, with some exceptions [36,37,38,39,40], they have suggested that during these periods, in SARS-CoV-2-infected women, pregnancy can be significantly different from and more complicated than that usually seen in noninfected women.

Unfortunately, no definitive conclusions can be drawn about the potential impact of SARS-CoV-2 infection on pregnancy during the first trimester. Few cases have been studied, and the methods used to perform some of the studies that have evaluated this problem are questionable and make the results debatable. However, it cannot be overlooked that the studies carried out during the first months of the pandemic have suggested that infection during an earlier stage of pregnancy does not cause fetal compromise or an increased risk of pregnancy loss [29,41,42,43,44,45,46]. The type and frequency of obstetric complications and the type and frequency of fetal and neonatal involvement seen in women infected during the last pregnancy periods varied significantly among the studies; in addition, there were differences in data collection, the characteristics of the pregnant women, concern for the pregnancy, iatrogenic interventions, the presence of a severe underlying disease, and the severity of COVID-19. Preterm delivery and stillbirth were the most frequently detected complications. Common, even if less frequent, were preeclampsia and caesarean section [47,48,49,50,51,52,53,54,55,56,57,58,59,60,61,62,63]. In a systematic review and meta-analysis of studies published until 29 January 2021, globally involving 7569 and 416,775 pregnant women with and without COVID-19, it was calculated that, compared with noninfected women, the odds ratios (OR) for preeclampsia, preterm birth and stillbirth were 1.33 (95% CI, 1.03–1.73), 1.82 (95% CI, 1.38–2.39), and 2.11 (95% CI, 1.14–3.90), respectively, in infected women [17]. The preterm birth incidence rate varied from 12.2% to 37% [58,59,60,61,62], compared to historical rates of 8.9–10.2% [30,59,60]. Finally, the risk of stillbirth was found to be more than five times higher in pregnant women with COVID-19 than in the general population (3.2% vs. 0.6%) [61]. The importance of the severity of maternal COVID-19 as a cause of complications is clearly highlighted by the evidence that patients with fever and shortness of breath had more complications (RR, 2.56; 95% CI, 1.92–3.40) and neonates with more clinical problems (RR, 4.97; 95% CI, 2.22–11.69) [16]. Moreover, in a large study involving cases collected in 13 US states, preterm birth was found to be three times more frequent in pregnant women with symptoms than in those without symptoms [62]. The same strict relationship with the severity of COVID-19 was evidenced for preeclampsia (OR, 4.16; 95% CI, 1.55–11.15) in the study by Wei et al. [17].

As expected, an increase in the severity of COVID-19 and obstetric complications was associated with neonatal complications. Two recent studies, both with a high number of enrolled pregnant women and with a low risk of bias, clearly highlight that neonates from infected women are at increased risk of clinical problems. In the Multicovid Multinational Cohort Study (16), the RR for low birth weight (<2500 g) was 1.58 (95% CI, 1.29–1.94), for a severe neonatal morbidity index (SNMI) was 2.66 (95% CI 1.69–4.18) and for a severe perinatal morbidity and mortality index (SPMMI) was 2.14 (95% CI 1.66–2.75) [17]. Definition of SNMI and SPMMI is summarized in Table 1.

In a Swedish prospective study in which 2323 children born to SARS-CoV-2-positive mothers were matched, directly and using propensity scores, on the basis of maternal characteristics with up to four comparator infants born to SARS-CoV-2-negative mothers, it was found that maternal COVID-19 was associated with a greater risk of admission for neonatal care (11.7% vs. 8.4%; OR 1.47; 95% CI, 1.26–1.70), respiratory distress syndrome (1.2% vs. 0.5%; OR, 2.40; 95% CI, 1.50–3.84, hyperbilirubinemia (3.6% vs. 2.5%; OR, 1.47; 95% CI, 1.13–1.90), and mortality (0.30% vs. 0.12%; OR, 2.55; 95% CI, 0.99–6.57) [18].

Although it seems certain that COVID-19 can complicate the course of pregnancy, the reasons why this occurs are not clear. However, some findings seem to suggest that most of the problems arise from the role directly played by how SARS-CoV-2 itself affects the cardiovascular and coagulation systems. Direct lesions can cause severe fetal vascular malperfusion and severe placental alterations that can significantly interfere with fetal health and development. In a study in which third-trimester placentas from 51 SARS-CoV-2-positive women and 25 controls were compared, placentas from infected women were more frequently characterized by villous agglutination (*p* = 0.003) and subchorionic thrombi (*p* = 0.026), although no evidence of direct viral involvement was identified [63]. Further support for the hypothesis of an autonomous role of SARS-CoV-2 in complication development is given by the evidence that the preeclampsia seen in pregnant women with COVID-19 is in some way different from the preeclampsia diagnosed in noninfected pregnant women. Both conditions have overlapping features, such as hypertension and thrombocytopenia, but in pregnant women with COVID-19, other preeclampsia markers, such as abnormally high levels of soluble fms-like tyrosine kinase-1 and the characteristic abnormal placentation, are lacking [64].

### 2.2. Transmission of SARS-CoV-2 from the Mother to the Child

As already detailed, most of the clinical problems that can develop in the fetus or in the neonate because of maternal SARS-CoV-2 infection depend on the impact of COVID-19 on pregnancy. However, at least theoretically, other mechanisms could be of relevance in this regard. SARS-CoV-2 can infect the placenta, interfering with fetal health [63,65,66,67,68,69]. Moreover, vertical transmission of SARS-CoV-2 from maternal blood to the fetus could occur [70,71,72,73,74,75,76,77,78,79,80,81,82,83,84,85,86,87,88]. Finally, the child could be infected by the mother after birth, during delivery or through breastmilk [89,90,91].

Regarding placental infection, several studies have shown that in pregnant women with COVID-19, SARS-CoV-2 can be detected in the placenta, mainly in the syncytio-trophoblast layer of the chorionic villi [63,65,66]. In some cases, the presence of the virus was associated with evidence of placental inflammation (20%), fetal vascular malperfusion (35.3%) and maternal vascular malperfusion (46%) [67]. Unfortunately, most of these studies have relevant limitations. Control groups are lacking. Moreover, in several cases, the studies were carried out in pregnant women infected during the third trimester of pregnancy, which does not allow us to understand whether placental alterations were dependent on direct viral placental damage or were simply the consequence of the vascular lesions that characterize COVID-19. On the other hand, the expression of human angiotensin-converting enzyme 2 (ACE2), essential for viral entry into cells, is low in the placenta [68], and the replication of the virus in placental cells has never been demonstrated [69]. Considering all these findings, it seems highly unlikely that placental infection by SARS-CoV-2 could play a relevant role in causing fetal and neonatal problems. However, as data in this regard are few and sometimes conflicting, further studies are needed to clarify the real importance of this complication and its relationship with gestational age and COVID-19 severity.

Regarding vertical transmission, most of the studies published to date seem to indicate that, if vertical transmission is possible, it is very rare. To occur, vertical transmission requires maternal viremia or the presence of SARS-CoV-2 in breast milk. However, studies have shown that maternal viremia is uncommon (no more than 10%) and transient [70,71]. Moreover, the detection of the virus in the blood/serum does not indicate virus infectivity. Andersson et al. reported that the inoculation of SARS-CoV-2 from the blood of infected individuals into cell culture did not produce any cytopathic effect or yield an increase in detectable SARS-CoV-2 RNA [72]. The search for viral RNA has been unsuccessful in the great majority of amniotic fluid, cord blood, newborn blood, cerebrospinal fluid, and meconium samples collected at delivery or in the first days after birth [31,73,74,75,76,77,78,79,80,81,82,83]. This has led most of the experts to conclude that the confirmation of vertical transmission requires the detection of viral RNAs in amniotic fluid prior to the onset of labour or in umbilical cord blood or neonatal blood within 12 h from birth [69]. The importance of SARS-CoV-2 detection in respiratory secretions without other confirmatory tests, despite the consideration of a potential case of vertical transmission [84], can be debated, as, in these cases, infection from sources other than the mother can be suggested. Doubts can also be raised in cases of positive neonatal serology, as IgM testing is poorly specific [85]. Following these criteria, true cases of confirmed or possible SARS-CoV-2 vertical transmission during fetal life remain very few. The estimated rate of 3%–8% suggested by some studies can be debated, as the criteria for vertical transmission used in these analyses were not stringent [86,87,88].

The transmission of SARS-CoV-2 during delivery or through breast milk can also be considered very rare [89]. Ascending infection has never been demonstrated, and vaginal delivery is not more common than caesarean section among children with suspected SARS-CoV-2 infection [69]. Regarding the risk of the transmission of SARS-CoV-2 through breastmilk, most of the studies have suggested that maternal feeding may not be a source of infection for the infant, as the virus was not detected in breastmilk collected during the first days after birth [70]. Moreover, when SARS-CoV-2 was evidenced by reverse transcriptase–polymerase chain reaction (RT-PCR), the viral culture for that sample was negative [90]. Finally, when the child developed mild symptoms of COVID-19 and had a positive RT-PCR SARS-CoV-2 diagnostic test, whether he was infected by breastfeeding or other modes of transmission remained unclear [91]. 

In conclusion, peri-natal vertical or horizontal transmission of SARS-CoV-2 is considered very uncommon. A recent systematic review of 26 studies, describing a total of 44 infected neonates, showed that the source of the infection remained unclear in most cases [92]. Diagnosis was mainly based on a positive nasopharyngeal, throat or anal swab collected 3–5 days from birth, and approximately half of the neonates could have been infected by family members or health caregivers [92]. However, even if the risk of transmission of SARS-CoV-2 from mother to child is very low or completely absent, further studies are needed to definitively establish the best management of babies born to SARS-CoV-2-infected mothers [93].

## 3. Prevention of COVID-19 in Pregnant Women with Vaccines

Studies in animals receiving COVID-19 vaccines presently authorized for emergency use in humans before or during pregnancy did not show safety problems in pregnant animals or offspring [14,94]. However, very few clinical trials studying the immunogenicity, safety and tolerability of COVID-19 vaccines in pregnant and lactating women and their impact on offspring have been planned, concluded and published. Most of the data available to date are for the two mRNA vaccines presently authorized for emergency use, the Pfizer-BioNTeck vaccine [95] and the Moderna vaccine [96]. Data about the nonreplicating viral vector vaccines, such as the AstraZeneca and Janssen preparations, are limited to those derived from studies specifically planned for phase III clinical trials in which pregnant women were inadvertently enrolled and are too few to allow conclusions [97]. Although adenoviral vectors do not seem to have a role anymore for vaccination during pregnancy because of safety issues, COVID-19 vaccines based on different technologies are in development and should be tested in pregnant women before their use.

### 3.1. Immunogenicity

The results of studies published until now show that mRNA COVID-19 vaccines can generate a robust humoral and cell-mediated response in pregnant and lactating women that is quite similar to that observed in nonpregnant women and is significantly superior to that evoked by SARS-CoV-2 infection [19,98,99]. Titers of IgG or IgA antibodies that bind to the SARS-CoV-2 spike receptor binding domain (RBD), serum antibodies that can neutralize viral particles and non-neutralizing antibodies were found to be several times higher in vaccinated women than in SARS-CoV-2-infected patients, regardless of whether they were pregnant, lactating, nonpregnant or nonlactating. Moreover, in immunized women, greater CD4 and CD8 T cell responses were evidenced, suggesting the induction of significantly higher immune memory. In the study by Colier et al. [98], 103 participants were enrolled. Among them, 30 were pregnant, 16 were lactating, and 57 were neither pregnant nor lactating. A total of 28 women infected by SARS-CoV-2, six of whom were pregnant, were included as controls. Blood samples for the immune study were withdrawn 21–26 days after the second vaccine dose and after a median of 12 and 41 days from symptom onset in infected nonpregnant and pregnant women, respectively. The median RBD–IgG binding antibody titers in immunized nonpregnant, pregnant, and lactating women were 37,839, 27,601, and 23,497, respectively, compared to 1321 and 771 in infected pregnant and nonpregnant women, respectively [98]. Similar differences were evidenced when the pseudo-virus neutralizing antibody titers were measured. In vaccinated nonpregnant pregnant and lactating subjects, titers were 901, 910 and 783, respectively, whereas they were 148 and 193 in infected pregnant and nonpregnant women, respectively [98]. Regarding the cellular response, the percentages of spike-specific interferon (IFN)-γ production by CD4 T cells, CD4 central memory T cells, CD8 T cells, and CD8 central memory T cells were comparable in pregnant, lactating, and nonpregnant women. 

The higher immune response of pregnant women to mRNA vaccines than of women with SARS-CoV-2 infection has a significant impact on cord-blood- and breastmilk-specific antibody titers, suggesting efficient transplacental antibody transfer and potential protection of the infant through maternal vaccination and breastfeeding [98,99,100]. In the cord blood of children born to vaccinated women, after the second vaccine dose, the median serum IgG binding antibody titers were at least half of those measured in maternal serum [98,99,100], whereas in breast milk, they were more than 15 times higher than those in infected subjects (25,055 after vaccination and 1593 after natural infection) [98]. A certain degree of protection through breastmilk could be achieved even after the first dose, as specific IgA antibodies could be detected in breastmilk just after the first mRNA vaccine dose [101]. The results of the more recent study by Gray et al. [99], which enrolled 84 pregnant, 31 lactating and 16 nonpregnant women and was carried out with similar methods, did not substantially differ from those reported by Collier et al. [98], confirming that mRNA vaccines might offer pregnant women the same protection already demonstrated in healthy nonpregnant individuals of the same age. Moreover, the immunization of pregnant women might be associated with significant reductions in the negative impact that maternal COVID-19 can have on pregnancy and on the conceptus. 

On the basis of these premises, pregnant women should be included in the priority list for COVID-19 vaccine administration, and mRNA vaccines should be authorized for emergency use in these individuals. However, further studies are needed. First, the effects of vaccines based on different conceptual and technological platforms must be established. Moreover, regarding mRNA vaccines, clinical trials with a low risk of bias including a greater number of pregnant women are needed to better characterize the immune response of these women and to establish the best schedule of administration to protect both the mother and the child. Moreover, the role of virus variants in pregnancy and measures to protect fetuses from these variants should be better defined, taking into account that in the study by Collier et al. [98], both immunized pregnant women and infant cord blood showed a significant reduction in neutralizing antibody titers against the B.1.1.7 variant (UK variant) and the B.1.351 variant (South Africa variant) [93]. Unfortunately, until the antibody levels that can be considered the correlate of protection are precisely established, these goals cannot be achieved.

### 3.2. Safety and Tolerability

To be recommended, a vaccine must be not only immunogenic but also tolerated and safe. Presently available data seem to indicate that the incidence and severity of solicited local and systemic reactions following the administration of mRNA vaccines to pregnant women are not substantially different from those occurring in nonpregnant women. The incidence of adverse events in the few pregnant women enrolled in the studies planned to evaluate the immunogenicity of mRNA vaccines suggests this conclusion [98,99]. However, more reliable and very reassuring information in this regard is derived from a large study carried out in the USA using data collected in 35,691 pregnant persons aged 16 to 54 years through two monitoring systems, the Vaccine Adverse Event Reporting System (VAERS), administered by the CDC and FDA, and the V-safe Surveillance System, a new CDC smartphone-based system specifically developed for the COVID-19 vaccination program [102]. Among immunized pregnant women, 19,252 (53.9%) and 16,439 (46.1%) had received the Pfizer-BioNTech vaccine and the Moderna vaccine, respectively. It was shown that injection-site pain, fatigue, headache, myalgia, chills and fever occurred in pregnant women with frequencies similar to those reported in nonpregnant women [102]. Moreover, in a group of 3958 women monitored by the V-safe Surveillance System, among whom 92 had received the vaccine during the periconception period, 1132 in the first trimester of pregnancy, 1714 in the second trimester and 1019 in the third trimester, incidence rates of spontaneous abortion (12.6%), preterm birth (9.4%) and small size for gestational age (3.2%) were not different from those reported in studies carried out in pregnant women before the onset of the COVID-19 pandemic [102].

Recently, an increased concern regarding COVID-19 vaccine and fertility has been reported as major cause for hesitancy in vaccine uptake [103]. Although no study has demonstrated an impact of SARS-CoV-2 infection and COVID-19 vaccination on ovarian function [104] or on sperm parameters [105], counselling with patients should be undertaken to help mitigate these concerns.

Safety and tolerability data regarding mRNA COVID-19 vaccines seem sufficiently satisfactory and adequate to support the authorization for the emergency use of these vaccines in pregnant women. However, because the study that strongly supports this conclusion has several limitations, further studies are needed. Problems emerge from the evidence that the results of this study cannot be directly compared with those of previous studies, as the study groups are not directly comparable, as these pregnant women differ in terms of social, demographic, and clinical characteristics. The surveillance systems used to collect the data used for this study have some limitations. The VAERS is a passive surveillance system, which can lead to underreporting, and reporting to both the VAERS and the V-safe Surveillance System is voluntary. Finally, the study included only a relatively small number of pregnant women who were carefully monitored, and most of them were vaccinated in the last part of the pregnancy. It cannot be excluded that, when studying a greater sample size and women at the beginning of pregnancy, different results could be obtained. Moreover, regarding vaccines based on different platforms, specific trials on each of them with appropriate sample size and adequate follow-up is needed before their use in clinical practice.

## 4. Conclusions

Table 2 summarizes the risks and benefits associated with COVID-19 and results obtained with COVID-19 vaccination in pregnancy. The results of studies carried out in the year following the declaration of the COVID-19 pandemic indicate that pregnant women are at increased risk of developing severe disease compared with nonpregnant women of the same age. As disease severity is associated with an increased risk of obstetric complications, children born to mothers with COVID-19 can have significant clinical problems because of prematurity, low weight for gestational age, and related diseases. Undetermined is the risk of vertical or horizontal mother-to-child transmission, although it cannot be excluded that some of the few cases of neonatal COVID-19 may have this origin. In addition, with the global dominance of the highly transmissible delta variant, it is vital that every eligible person be vaccinated to prevent the further spread and mutation of SARS-CoV-2. All these findings strongly suggest that COVID-19 vaccines should be administered to pregnant women. 

The results of studies carried out with mRNA vaccines indicate that the immunogenicity, safety and tolerability of these preventive measures in pregnant women are not different from those in nonpregnant women of the same age. Moreover, antibodies are efficiently transferred through the placenta and can be detected in breastmilk, suggesting a potential prevention of infection in the child. All these findings authorize the use of mRNA vaccines in pregnant women to protect both the mother and the child. However, further studies with larger sample size and with follow-up of the pregnant women vaccinated during different periods of pregnancy and their children are needed to better characterize the immune response of pregnant women, to define when these vaccines should be administered to obtain the best protection, and to measure vaccine efficacy against virus variants in both mothers and infants. COVID-19 vaccines based on different technological platforms cannot presently be used, and their role in pregnant women should be clarified.

## Figures and Tables

**Table 1:** Definition of severe neonatal mortality index (SNMI) and severe perinatal morbidity and mortality index (SPMMI).

Index	Criteria
SNMI	At least 3 of the following severe complications: bronchopulmonary dysplasia, hypoxic-ischaemic encephalopathy, sepsis, anaemia requiring transfusion, patent ductus arteriosus requiring treatment or surgery, intraventricular haemorrhage, and necrotizing enterocolitis or retinopathy of prematurity diagnosed before hospital discharge.
SPMMI	Any of the morbidities listed in the SNMI or intrauterine or neonatal death or neonatal ICU stay for 7 days

ICU, intensive care unit.

**Table 2:** Risks and benefits associated with COVID-19 and results obtained with COVID-19 vaccination in pregnancy.

Issue	Main Results
COVID-19 severity	Increased risk of severe COVID-19 in pregnant women (i.e., hospitalization, admission to the intensive care unit, ventilatory support and death)
Pregnancy complications	Increased risk of preterm delivery and stillbirth, preeclampsia and caesarean section
Neonatal complications	Low birth weight (<2500 g) Severe neonatal morbidity index (SNMI) Severe perinatal morbidity and mortality index (SPMMI)
Transmission of SARS-CoV-2 from the mother to the child	Undetermined risk of vertical transmission Transmission by the mother after birth during delivery or through breastmilk
Immunogenicity of mRNA vaccines against COVID-19	Similar immunogenicity in pregnant women and in nonpregnant women of the same age Specific antibodies efficiently transferred through the placenta and detected in breastmilk
Safety and tolerability of mRNA vaccines against COVID-19	Similar safety and tolerability in pregnant women and in nonpregnant women of the same age No impact on females’ and males’ fertility

## Data Availability

Not applicable.
